# Anticancer Potential of Prebiotics: Targeting Estrogen Receptors and PI3K/AKT/mTOR in Breast Cancer

**DOI:** 10.3390/biomedicines13040990

**Published:** 2025-04-18

**Authors:** Hussein Sabit, Sama Abouelnour, Bassel M. Hassen, Salma Magdy, Ahmed Yasser, Al-Hassan Soliman Wadan, Shaimaa Abdel-Ghany, Faisal Radwan, Amany I. Alqosaibi, Hala Hafiz, Ohaad F. A. Awlya, Borros Arneth

**Affiliations:** 1 Department of Medical Biotechnology, College of Biotechnology, Misr University for Science and Technology, Giza P.O. Box 77, Egypt; 2 Department of Environmental Biotechnology, College of Biotechnology, Misr University for Science and Technology, Giza P.O. Box 77, Egypt; 3 Department of Agri-Biotechnology, College of Biotechnology, Misr University for Science and Technology, Giza P.O. Box 77, Egypt; 4 Oral Biology Department, Faculty of Dentistry, Galala University, Galala Plateau, Attaka, Suez Governorate 15888, Egypt; alhassan.soliman.168@gmail.com; 5 Center for Coastal Environmental Health and Biomolecular Research, NCCOS/NOS/NOAA, Charleston, SC 29412, USA; 6 Department of Biology, College of Science, Imam Abdulrahman bin Faisal University, P.O. Box 1982, Dammam 31441, Saudi Arabia; 7 Clinical Nutrition Department, Faculty of Applied Medical Sciences, Umm Al-Qura University, P.O. Box 715, Makkah 21955, Saudi Arabia; 8 Institute of Laboratory Medicine and Pathobiochemistry, Molecular Diagnostics, Hospital of the Universities of Giessen and Marburg (UKGM), Philipps University Marburg, Baldingerstr. 1, 35043 Marburg, Germany; 9 Institute of Laboratory Medicine and Pathobiochemistry, Molecular Diagnostics, Hospital of the Universities of Giessen and Marburg (UKGM), Justus Liebig University Giessen, Feulgenstr. 12, 35392 Giessen, Germany

**Keywords:** estrogen receptors, breast cancer, prebiotics, gut microbiota, short-chain fatty acids

## Abstract

Estrogen receptors (ERs) play a critical role in breast cancer (BC) development and progression, with ERα being oncogenic and ERβ exhibiting tumor-suppressive properties. The interaction between ER signaling and other molecular pathways, such as PI3K/AKT/mTOR, influences tumor growth and endocrine resistance. Emerging research highlights the role of prebiotics in modulating gut microbiota, which may influence estrogen metabolism, immune function, and therapeutic responses in BC. This review explores the impact of prebiotics on estrogen receptor modulation, gut microbiota composition, immune regulation, and metabolic pathways in breast cancer. The potential of prebiotics as adjunctive therapies to enhance treatment efficacy and mitigate chemotherapy-related side effects is discussed. A comprehensive analysis of recent preclinical and clinical studies was conducted, examining the role of prebiotics in gut microbiota modulation, immune regulation, and metabolic reprogramming in breast cancer. The impact of short-chain fatty acids (SCFAs) derived from prebiotic fermentation on epigenetic regulation and endocrine resistance was also evaluated. Prebiotics were found to modulate the gut microbiota-estrogen axis, reduce inflammation, and influence immune responses. SCFAs demonstrated selective estrogen receptor downregulation and metabolic reprogramming, suppressing tumor growth. Synbiotic interventions mitigate chemotherapy-related side effects, improving the quality of life in breast cancer patients. Prebiotics offer a promising avenue for breast cancer prevention and therapy by modulating estrogen metabolism, immune function, and metabolic pathways. Future clinical trials are needed to validate their efficacy as adjunctive treatments in breast cancer management.

## 1. Introduction

Breast cancer remains the most prevalent malignancy among women worldwide, with its incidence rising steadily over the past decades [1,2]. Advances in molecular profiling have uncovered key oncogenic pathways, including the role of tumor microenvironment components such as senescent cancer-associated fibroblasts in driving immune evasion and tumor progression [3]. Therapeutic developments, such as CDK4/6 inhibitors ribociclib and palbociclib, have significantly improved survival outcomes in hormone receptor-positive HER2-negative breast cancer [4]. Additionally, lipid metabolism dysregulation, particularly alterations in phospholipids and sphingolipids, has been implicated in breast cancer progression and response to therapy [5]. Precision medicine approaches integrating genomic and metabolomic insights are reshaping treatment strategies, offering targeted therapies such as PI3K inhibitors and immune checkpoint blockade [6,7]. However, persistent disparities in outcomes, particularly among racial and ethnic minorities, highlight the urgent need for equitable access to screening and personalized interventions [1]. Future research should focus on refining immunometabolic targets, improving survivorship care, and integrating real-time molecular monitoring to enhance treatment efficacy and patient outcomes [8,9].

The phosphoinositide 3-kinase (PI3K)/Akt/mTOR pathway is a key oncogenic driver, implicated in tumorigenesis, metastasis, and therapy resistance, particularly in hormone receptor-positive (HR+) and triple-negative breast cancer (TNBC) [10,11]. Dysregulation of this pathway contributes to enhanced proliferation, epithelial–mesenchymal transition, and immune evasion, underscoring the need for targeted interventions [12]. Novel approaches, including CDK4/6 and PI3K/mTOR inhibitors, offer combinatorial benefits in HR+/HER2– breast cancer, yet resistance mechanisms necessitate further exploration of alternative signaling cascades and immune-modulating strategies [13,14]. As precision medicine evolves, integrating genomic, metabolomic, and immunotherapeutic insights will be critical to optimizing patient outcomes and overcoming therapeutic resistance [9].

The human gut microbiota has emerged as a critical regulator of health and disease, influencing metabolism, immunity, and neurological function [15,16].

Prebiotics, primarily fermentable dietary fibers, promote the growth of beneficial gut bacteria, leading to the production of short-chain fatty acids (SCFAs) that exert systemic metabolic and immunomodulatory effects [17,18]. Recent studies highlight the impact of prebiotics and probiotics on conditions such as inflammatory skin diseases [19], metabolic disorders [20], and mental health disorders, including depression [21]. The gut–liver and gut–brain axes have been increasingly recognized as important mediators of these effects, with evidence suggesting that dietary interventions targeting the gut microbiota could play a role in disease prevention and therapy [22]. Beyond general health benefits, prebiotics also show promise in cancer prevention and treatment, particularly in colon and breast cancer, by modulating immune responses and inhibiting tumor growth [23,24]. The enrichment of SCFA-producing bacteria through prebiotic supplementation has been linked to reduced systemic inflammation, improved lipid metabolism, and enhanced insulin sensitivity, influencing the progression of metabolic and hormone-related cancers [25]. Furthermore, specific prebiotic compounds have been shown to enhance calcium absorption and bone mineralization, which is crucial for patients at risk of osteoporosis due to cancer-related treatments [26]. Despite these promising findings, further clinical trials and mechanistic studies are needed to fully understand the therapeutic potential of prebiotics in various disease contexts, including oncology, metabolic syndrome, and immune disorders [17].

This study aims to comprehensively explore the interplay between estrogen receptor (ER) signaling and the PI3K/AKT/mTOR pathway in breast cancer, emphasizing its role in tumor progression, endocrine resistance, and therapeutic targeting. Additionally, we evaluate the emerging role of prebiotics in modulating gut microbiota, estrogen metabolism, and immune function, highlighting their potential as adjunctive therapies to enhance breast cancer treatment outcomes and reduce therapy resistance.

## 2. Estrogen Receptor Signaling in Breast Cancer

### 2.1. Role of Estrogen Receptors in BC Development and Progression

Estrogen receptors (ERs), particularly estrogen receptor alpha (ERα), play a crucial role in breast cancer development, progression, and treatment. ERα is a key oncogene in breast cancer, driving tumor growth and progression in approximately 70–80% of breast tumors [27]. The activation of ER signaling through estrogen binding promotes cell proliferation, differentiation, and survival in hormone-sensitive cancers [28]. Estrogen receptors (ERs) are ligand-activated transcription factors that play crucial roles in gene regulation and exhibit both overlapping and specific tissue distribution patterns. The two main types of nuclear estrogen receptors are ERα and ERβ, which have distinct functions and distributions in breast tissue [29]. ERα is predominantly expressed in a subset of luminal cells, corresponding to less than 10% of normal mammary epithelial cells. However, it is present in over 70% of breast tumors, making it a key driver of tumorigenesis in breast cancer, especially in the presence of estrogen [30]. ERα is generally considered oncogenic and is associated with the proliferation of breast cancer cells, especially in the presence of estrogen [31].

In contrast, ERβ has been shown to act as an oncosuppressor in several instances, although its exact role in carcinogenesis and tumor progression is not yet fully understood. ERβ is expressed in a variety of breast cancers, including triple-negative breast cancers (TNBCs), which has led to its consideration as a potential target for cancer therapy [29].

Interestingly, the distribution and functions of ERα and ERβ in breast tissue can be influenced by various factors. For instance, matrix stiffness has been found to play a crucial role in regulating ERα expression and signaling in breast tissue. Increased matrix stiffness upregulates ERα signaling through stress-mediated p38 activation and H3K27me3-mediated epigenetic regulation [32]. This mechanobiological component adds another layer of complexity to the understanding of estrogen receptor distribution and function in breast tissue.

### 2.2. Mechanisms of Endocrine Resistance and Targeted Therapies

The importance of ERs in breast cancer is underscored by their role as predictive biomarkers and therapeutic targets. ER-positive breast cancers are generally more responsive to endocrine therapies, which aim to inhibit estrogen signaling through various mechanisms [33]. However, the development of resistance to these therapies remains a significant challenge in breast cancer treatment.

**Figure 1:**
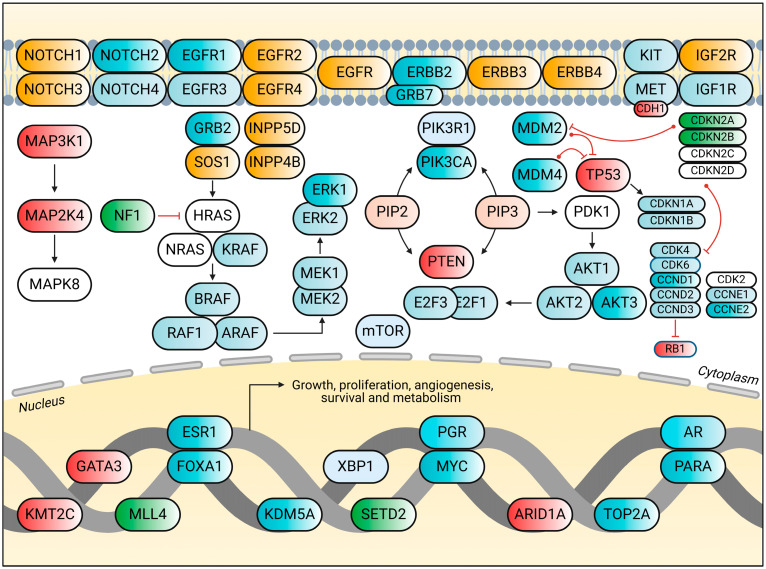
The molecular signaling pathways involved in breast cancer. The key gene mutations and their interactions are highlighted. The diagram shows the involvement of receptor tyrosine kinases (e.g., *EGFR, ERBB* family), signaling molecules (e.g., *PIK3CA, AKT, MAPK*), tumor suppressors (e.g., *PTEN*, *TP53*), and cell cycle regulators (e.g., *CDKN2A*, *RB1*). These genes and proteins contribute to crucial cellular processes such as growth, proliferation, survival, and metabolism in breast cancer. Notably, alterations in these pathways can lead to cancer progression, highlighting their importance in therapeutic targeting.

Recent research has uncovered complex molecular mechanisms underlying ER signaling in breast cancer (Figure 1). For instance, transcription factors such as ETV4 have been found to control ER genomic binding and contribute to estrogen signaling [34]. Additionally, post-translational modifications, such as ubiquitination, play a crucial role in regulating ER protein stability and activity [35,36]. Understanding these mechanisms is essential for developing more effective therapeutic strategies and overcoming endocrine resistance in breast cancer.

Estrogen receptor (ER) mutations, particularly in the *ESR1* gene, play a significant role in breast cancer development and treatment resistance. These mutations have been identified as drivers of resistance and disease recurrence in hormone receptor-positive breast cancer patients [37,38]. Approximately 40% of breast cancer patients develop resistance to endocrine therapy, with *ESR1* mutations being a common mechanism of acquired resistance [39].

*ESR1* mutations can lead to constitutively active forms of the estrogen receptor, which remain functional even in the absence of estrogen [40]. This alteration in ER signaling can render traditional endocrine therapies, such as aromatase inhibitors and tamoxifen, less effective. The discovery of these mutated forms of ERα in metastatic hormonotherapy-resistant breast cancer has provided a strong rationale for developing new antiestrogens targeting clinically relevant ERα mutants [41,42].

To address the challenge posed by *ESR1* mutations, researchers have developed selective estrogen receptor degraders (SERDs). The first-generation SERD, fulvestrant, shows activity against *ESR1* mutant tumors. However, its poor bioavailability and need for intramuscular injections limit its effectiveness. Second-generation oral SERDs with improved bioavailability and pharmacokinetics are now in phase III trials for both early and advanced ER-positive breast cancer [43]. These new SERDs, such as palazestrant (OP-1250), demonstrate superior performance in wild-type and *ESR1*-mutant breast cancer models compared to the existing treatments [44]. Additionally, combination therapies targeting multiple pathways, such as CDK4/6 inhibitors or PI3K inhibitors, are being explored to overcome resistance mechanisms [45].

### 2.3. Crosstalk Between ER Signaling and Other Pathways in BC

Estrogen receptor (ER) signaling is a critical regulator of cell proliferation, differentiation, and survival in breast cancer. Estrogens act as growth factors necessary for cancer invasion and dissemination [28].

Interestingly, ER signaling interacts with other pathways to promote cancer progression. For instance, crosstalk between classical (ER and PR) and nonclassical (PGRMC1) signaling mechanisms exists in ER+ breast cancer cells, enhancing the growth of ER+/PR+/PGRMC1-overexpressing tumors [46]. Additionally, ER regulates the miR-29a–PTEN–AKT axis, which governs breast cancer progression and metastasis. High levels of miR-29a are associated with distant metastasis and poor survival in triple-negative breast cancer [47].

The PI3K/AKT/mTOR signaling pathway plays a central role in regulating cellular functions such as growth, proliferation, and survival. In breast cancer, this pathway is frequently hyperactivated, contributing to tumor progression and resistance to various therapies [48,49,50]. Dysregulation of the PI3K/AKT/mTOR pathway is particularly prevalent in hormone receptor-positive (HR+) breast cancers, where it is associated with endocrine therapy resistance [51].

Interestingly, the polyamine pathway has been found to interact with the PI3K/AKT/mTOR pathway in breast cancer cells. Modulation of intracellular polyamine levels affects downstream targets of mTORC1, such as 4EBP1 and p70S6K phosphorylation, suggesting a potential link between these two pathways in promoting cancer cell growth [52,53]. Additionally, the nuclear envelope component PRR14 has been identified as a novel interacting node between the PI3K/AKT/mTOR and CHEK2 pathways, contributing to breast carcinogenesis and chemotherapy resistance [54].

The crosstalk between estrogen receptor (ER) signaling and other important pathways in breast cancer, such as PI3K/AKT, plays a crucial role in tumor growth, progression, and resistance to therapy. As we mentioned above, the PI3K/AKT/mTOR pathway is particularly significant in the context of endocrine resistance. This pathway is frequently activated in ER-positive breast cancers and plays a crucial role in sustaining resistance to ER-targeted therapies [55].

**Figure 2:**
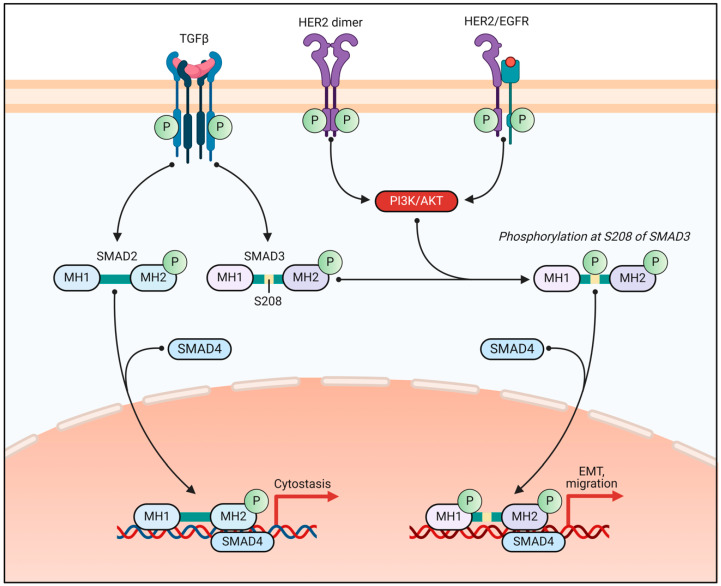
The role of PI3K/AKT signaling in modulating the TGF-β/SMAD3 pathway in breast cancer progression. This schematic illustrates the crosstalk between TGF-β and HER2/EGFR signaling in breast cancer cells. TGF-β signaling leads to the phosphorylation of SMAD2 and SMAD3, enabling their complex formation with SMAD4, which translocates to the nucleus. Unmodified SMAD3, in association with SMAD4, induces cytostasis (cell cycle arrest). However, HER2 and EGFR signaling, through activation of the PI3K/AKT pathway, leads to phosphorylation of SMAD3 at serine 208 (S208), altering its function. This modified SMAD3, in complex with SMAD4, promotes epithelial-to-mesenchymal transition (EMT) and migration, which are hallmarks of cancer progression and metastasis. This pathway highlights a potential mechanism by which HER2-driven breast cancer evades TGF-β-induced tumor suppression and instead promotes tumor progression.

Given the importance of the PI3K/AKT/mTOR pathway in breast cancer, numerous therapeutic strategies targeting this pathway have been developed. These include PI3K inhibitors, mTOR inhibitors, and dual PI3K/mTOR inhibitors [50]. Novel approaches, such as nanozyme-based therapies targeting PI3K subunits, have shown promise in overcoming tamoxifen resistance [56]. Additionally, natural compounds such as myo-inositol have demonstrated potential in inhibiting the PI3K/AKT pathway and suppressing metastasis in triple-negative breast cancer cells [57]. As research continues, targeting specific components of the pathway, such as mTORC2 and its partner SIN1, may provide more effective and less toxic therapeutic options for breast cancer patients [58] (Figure 2).

Beyond intracellular signaling pathways, emerging evidence highlights the influence of systemic factors—such as the gut microbiota—on breast cancer behavior and therapeutic efficacy. In this context, modulation of host–microbiota interactions are gaining attention as a complementary strategy to enhance treatment outcomes in ER⁺ breast cancer.

## 3. The Anticancer Potential of Prebiotics

Breast cancer remains the most prevalent cancer among women, accounting for approximately 25% of all newly diagnosed cases and 16% of global cancer-related deaths [59]. In 2023, an estimated 300,000 new cases of invasive breast cancer were projected in the United States, with approximately 43,700 deaths [60]. Despite significant advances in treatment, prevention strategies and adjunct therapies are urgently needed to improve patient outcomes. One emerging avenue is the role of diet and the gut microbiome in modulating cancer risk and therapy response [61,62].

Prebiotics—indigestible dietary fibers that selectively promote beneficial gut bacteria—have attracted interest for their potential anticarcinogenic effects in breast cancer [63]. Research has shown that gut microbiota influences systemic estrogen levels, immune regulation, inflammation, and even the efficacy of cancer therapies [64,65]. Preclinical studies suggest that fermentable fiber-enriched diets can reduce mammary tumor growth and enhance therapy response through short-chain fatty acid (SCFA) production, modulation of estrogen metabolism, and improved immune surveillance [66,67]. Epidemiological analyses also correlate higher dietary fiber intake with a lower risk of breast cancer [68].

### 3.1. The Microbiota–Estrogen Axis

The interplay between gut microbiota and estrogen metabolism is critical in hormone-dependent cancers [69]. The estrobolome refers to the collection of microbial genes capable of metabolizing estrogens [70]. By influencing the enterohepatic circulation of estrogens, the estrobolome can impact systemic estrogen exposure and estrogen receptor α-positive (ER⁺) breast cancer growth [71].

The liver metabolizes estrogens (estradiol, estrone, and estriol) and excretes them as conjugated metabolites into bile. Gut microbiota with β-glucuronidase enzymes can deconjugate these metabolites, allowing them to be reabsorbed into circulation [72]. Dysbiosis with elevated β-glucuronidase activity—often linked to certain *Clostridium* and *Escherichia* species—can contribute to higher systemic estrogen levels and increased ER-driven tumor growth. Conversely, beneficial microbes such as *Lactobacillus* and *Bifidobacterium* have lower β-glucuronidase activity and can suppress estrogen reabsorption [69].

Prebiotics (e.g., inulin, fructooligosaccharides, and galactooligosaccharides) selectively enhance beneficial gut bacteria that modulate estrogen metabolism. Clinical trials demonstrate that prebiotic consumption increases *Bifidobacterium* levels in the gut, leading to reduced fecal β-glucuronidase activity and lower circulating estrogen [73,74,75]. Experimental studies show that prebiotic-rich diets shift gut microbial composition, reduce serum estradiol, and slow estrogen-dependent tumor growth [72].

SCFAs produced from prebiotic fermentation create an acidic colonic environment that inhibits β-glucuronidase-producing bacteria, further reducing estrogen reabsorption [76]. High-fiber diets may also influence estrogen levels via weight regulation, as excess adipose tissue is a major estrogen source in postmenopausal women. Meta-analyses report that high fiber intake correlates with an 8–12% reduction in breast cancer risk, partly due to lowering body weight and estrogen production [77].

### 3.2. SCFAs and Epigenetic Reprogramming

Prebiotics exert additional anticancer effects through the microbial production of SCFAs, including acetate, propionate, and butyrate [78]. SCFAs act as natural HDAC inhibitors, altering chromatin structure and gene expression. Notably, butyrate downregulates ERα expression, mimicking the effects of selective estrogen receptor degraders (SERDs) such as fulvestrant [79].

SCFAs also inhibit key oncogenic pathways, including the PI3K/AKT/mTOR and NF-κB signaling cascades, thereby reducing tumor growth and survival [80]. Butyrate has been shown to induce apoptosis in breast cancer cells through mitochondrial disruption, oxidative stress, and G₁ cell cycle arrest [81]. Additionally, SCFAs can enhance sensitivity to hormonal treatments, as shown in studies where butyrate combined with DNA methyltransferase inhibitors improved response rates in ER⁺ breast cancer cells [82].

SCFAs influence breast cancer cell metabolism by disrupting the Warburg effect—aerobic glycolysis commonly seen in tumors [83]. Butyrate downregulates glucose transporters such as GLUT1, limiting cancer cell glucose uptake and ATP production [84]. Furthermore, SCFAs inhibit fatty acid synthase (FAS) and acetyl-CoA carboxylase (ACC), reducing lipid synthesis necessary for tumor cell membrane production [85]. This metabolic shift creates selective pressure against cancer cells while allowing normal cells to thrive [86].

### 3.3. Anti-Inflammatory Roles of Prebiotics in BC

The gut microbiota plays a crucial role in shaping immune function, which directly impacts breast cancer progression [62]. SCFAs modulate the activity of innate immune cells, promoting tumoricidal macrophages (M1) while suppressing tumor-associated macrophages (M2) [87]. Butyrate has also been shown to enhance natural killer (NK) cell cytotoxicity, contributing to improved immune surveillance [88].

Chronic inflammation contributes to breast cancer initiation and progression. Pro-inflammatory cytokines such as TNF-α and IL-6 can promote estrogen metabolism into DNA-damaging metabolites and activate NF-κB signaling, which drives tumor proliferation [89,90]. Prebiotics help mitigate inflammation through SCFA-mediated inhibition of NF-κB, reducing cytokine production [91].

**Figure 3:**
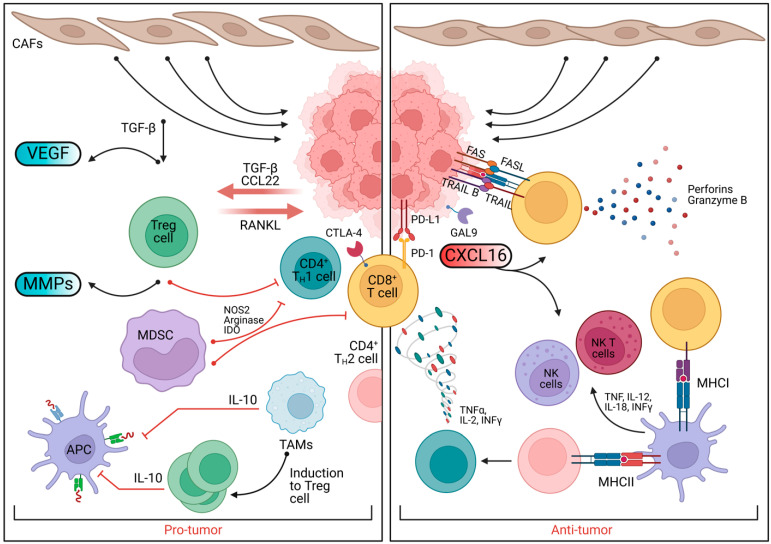
The contrasting roles of protumor and antitumor immune responses in the tumor microenvironment. On the left (protumor), various immune cells, such as Treg cells, MDSCs, and TAMs, contribute to tumor progression through mechanisms such as the induction of Treg cells, secretion of IL-10, and suppression of immune responses. Factors such as VEGF, MMPs, and RANKL support tumor growth and immune evasion. On the right (antitumor), immune cells such as CD8+ T cells, NK cells, and APCs activate antitumor responses, with cytokines such as TNF-α, IL-2, and IFN-γ promoting immune attack. The interaction of CXCL16 with immune cells enhances the tumor-killing activity of CD8+ T cells and NK cells. The balance between these pro- and antitumor mechanisms is critical for tumor progression and immune therapy responses.

Moreover, high-fiber diets expand regulatory T cell (Treg) populations, producing IL-10, an anti-inflammatory cytokine that protects against DNA damage and protumor signaling [92,93]. Prebiotics also enhance Th1 immune responses, increasing levels of IFN-γ and IL-12, which stimulate cytotoxic T cells and macrophages that target tumors [94] (Figure 3).

Prebiotics exhibit promising anticancer properties in breast cancer prevention and therapy through multiple mechanisms. They modulate estrogen metabolism by shaping the gut microbiota, reduce tumor-promoting inflammation, and influence cancer cell metabolism through SCFA production. Emerging research suggests that prebiotics may enhance the efficacy of endocrine and metabolic therapies, presenting an exciting avenue for integrative cancer treatment. Further clinical trials are needed to validate these findings and optimize prebiotic-based interventions for breast cancer patients.

### 3.4. Dietary Interventions Targeting mTOR in ER+ BC

Recent studies demonstrate how a specific diet can reduce leucine levels in the tumor microenvironment, thereby inhibiting mTORC1 activation [95]. SCFAs, derived from resistant starch as prebiotics, can induce autophagy in cancer cells by downregulating mTOR signaling [96].

Similarly, inulin supplementation has been associated with changes in gut microbial composition, leading to increased production of short-chain fatty acids, such as propionic acid, which can influence cancer cell proliferation by modulating mTOR signaling [81]. Notably, pentadecanoic acid also revealed a unique capacity to restore ERα expression in treatment-resistant cells while concurrently suppressing mTOR activity. This combined action resensitized resistant tumors to tamoxifen therapy [97] (Table 1).

## 4. Evidence from Preclinical and Clinical Studies

It was reported that giving young mice a diet with broccoli sprouts plus green tea polyphenols led to a significant inhibition of mammary tumor growth; analysis showed an increase in SCFA-producing taxa and beneficial genera such as *Lactococcus* and *Ruminococcaceae* in the gut of those mice [66].

A 2022 study found that combining exercise and inulin fiber in breast cancer survivor mice altered their gut microbiota, reducing inflammation and slowing tumor growth in recipient germ-free mice. These benefits were linked to increased SCFAs (butyrate/propionate) and improved gut barrier function [98].

Another study on 1958 women found that higher fiber consumption correlated with lower plasma levels of interleukin 6 (IL-6) and tumor necrosis factor-α receptor-2 (TNF-α-R2), though not C-reactive protein [118]. Obesity is associated with modifications in adipokine and cytokine profiles, characterized by elevated levels of leptin and diminished levels of adiponectin, which may facilitate the progression of breast cancer [119].

In women with obesity, weight reduction achieved through dietary interventions has been demonstrated to diminish the levels of pro-inflammatory markers. A 12-week dietary intervention study involving 29 premenopausal obese women showed significant reductions in weight, body mass index, and plasma levels of IL-6 and IL-18 [120].

### 4.1. Prebiotics: Systemic Health

The gut–brain axis is a bidirectional communication system between the gastrointestinal tract and the central nervous system, regulated through neural, endocrine, immune, and microbial metabolite pathways. The gut microbiota plays a pivotal role in modulating neurotransmitter synthesis, neuroinflammation, blood–brain barrier (BBB) integrity, and cognitive function [121].

Novel research highlights the safeguarding properties of butyrate, a short-chain fatty acid produced by intestinal microbiota, concerning the structural integrity of the blood–brain barrier (BBB). Butyrate strengthens tight junction proteins, particularly occludin and claudin-5, in brain endothelial cells, preventing neurotoxic molecules from entering the brain [122].

It has anti-inflammatory properties, contributing to the decrease in neuroinflammation, a common aspect noted in neurodegenerative ailments such as Alzheimer’s disease (AD). Furthermore, it helps in the formation of the brain-derived neurotrophic factor (BDNF), a significant protein that supports the preservation, growth, and transformation of neurons, consequently affecting mental functions and possibly reducing cognitive deterioration [123].

SCFAs can cross the blood–brain barrier and interfere with amyloid-β (Aβ) peptide aggregation, reducing the formation of neurotoxic oligomers [124]. Long-term dietary SCFA supplementation in AD mouse models has been found to alleviate cognitive impairment by reducing Aβ deposition and tau hyperphosphorylation. SCFAs also promote the glutamate–glutamine shuttle between astrocytes and neurons, potentially enhancing neuroprotection against oxidative damage [99].

Acetate and propionate can cross the BBB and influence brain function by modulating neurotransmitter systems. They are involved in the synthesis of neurotransmitters, primarily gamma-aminobutyric acid (GABA) and serotonin, which are important for emotional changes and aiding cognitive functions [125].

In addition, SCFAs have been shown to have neuroprotective effects, particularly in pediatric populations, by influencing immune responses and maintaining gut barrier integrity [126]. In experimental models, SCFAs have demonstrated the ability to mitigate neuroinflammation and protect against neurodegenerative processes, such as those seen in Alzheimer’s disease and Japanese encephalitis [127,128].

Chronic mild gut inflammation accelerates α-synuclein aggregation and motor dysfunction in Parkinson’s disease (PD) mouse models [129]. The gut–brain axis is implicated as a key pathological origin and potential therapeutic target for PD [130]. Prebiotic interventions show promise in modulating PD symptoms. A fiber-rich diet attenuates motor deficits, reduces α-synuclein aggregation, and promotes beneficial microglial states in α-synuclein-overexpressing mice [131].

Beyond the systemic effects of prebiotics on neurological functions, they also have a cardiological impact in patients diagnosed with breast cancer, who are already exhibiting heightened susceptibility to cardiovascular complications attributable to the cardiotoxic effects of chemotherapy, alongside inflammation and metabolic alterations. Prebiotics and gut microbiota modulation have been shown to support heart health through lipid regulation, blood pressure control, endothelial function, and inflammation reduction [132].

Intriguingly, SCFA administration, such as propionate, lowers vascular dysfunction and heart hypertrophy in hypertensive mice. The administration of inulin has been shown to reduce cardiometabolic risk and enhance antioxidant capacity in women diagnosed with diabetes. It was reported to lower systolic blood pressure (SBP) and decrease elevations in diastolic blood pressure (DBP) and MAP in breast cancer patients receiving neoadjuvant therapy in several studies that used double-blind, placebo-controlled trials [133].

Butyrate, a short-chain fatty acid, has been demonstrated to reduce blood pressure via multiple physiological mechanisms. It activates GPR41/43 receptors, leading to vasodilation and reduced hypertension [134]. GPR41/43 signaling is crucial for maintaining gut epithelial barrier integrity, preventing bacterial toxin translocation, and reducing renal inflammation. Lack of these receptors increases the risk of hypertension and cardiorenal fibrosis [135].

Sodium butyrate supplementation during pregnancy and lactation can prevent hypertension in offspring exposed to a maternal tryptophan-free diet by modulating gut microbiota and restoring the renin–angiotensin system balance [136]. SCFAs, particularly acetate and butyrate, enhance nitric oxide (NO) production and bioavailability, promoting vasodilation and improving endothelial function [137].

### 4.2. Preclinical Studies

#### 4.2.1. Breast Microbiota and Tumor Biology

Investigations into the breast microbiome have elucidated distinct microbial profiles in both normative and neoplastic tissues. A particular study indicated that *Lactobacillaceae, Acetobacteraceae,* and *Xanthomonadaceae* primarily inhabit normal breast tissue, whereas *Ralstonia* demonstrated greater prevalence in breast tumors and adjacent normal tissues [138]. Importantly, these discrepancies in microbial composition were correlated with variations in gene expression patterns. Normal tissues displayed a heightened activity in the genes associated with metabolism and immune functions, whereas the presence of *Ralstonia* was related to perturbations in carbohydrate metabolism.

Furthermore, an additional study assessing microbiota–immune interactions in both benign and cancer-affiliated breast tissues discovered that benign tissues possessed a more cohesive microbial network. Key contributors to this network included *Anaerococcus*, *Caulobacter*, and *Streptococcus*, which were conspicuously absent in cancerous tissues. The composition of these microbes exerted a significant influence on the expression of immune-related genes, for instance, *Propionibacterium* and *Staphylococcus*, which were diminished in tumors and exhibited a negative correlation with oncogenic immune characteristics, while *Streptococcus* and *Propionibacterium* demonstrated a positive correlation with genes associated with T cell activation [139].

#### 4.2.2. Gut Microbiota and Breast Cancer Prognosis

The gut microbiota has emerged as a key player in shaping the prognosis of early breast cancer and mediating chemotherapy-related side effects. A clinical study utilizing shotgun metagenomics revealed that breast cancer patients exhibit an altered gut microbial composition compared to healthy individuals. These differences were associated with clinical parameters such as tumor size and lymph node involvement. Moreover, specific gut bacteria that were overrepresented in breast cancer patients negatively impacted disease prognosis, suggesting the potential utility of microbiota monitoring as a predictive tool for treatment efficacy and side effect management [140].

**Figure 4:**
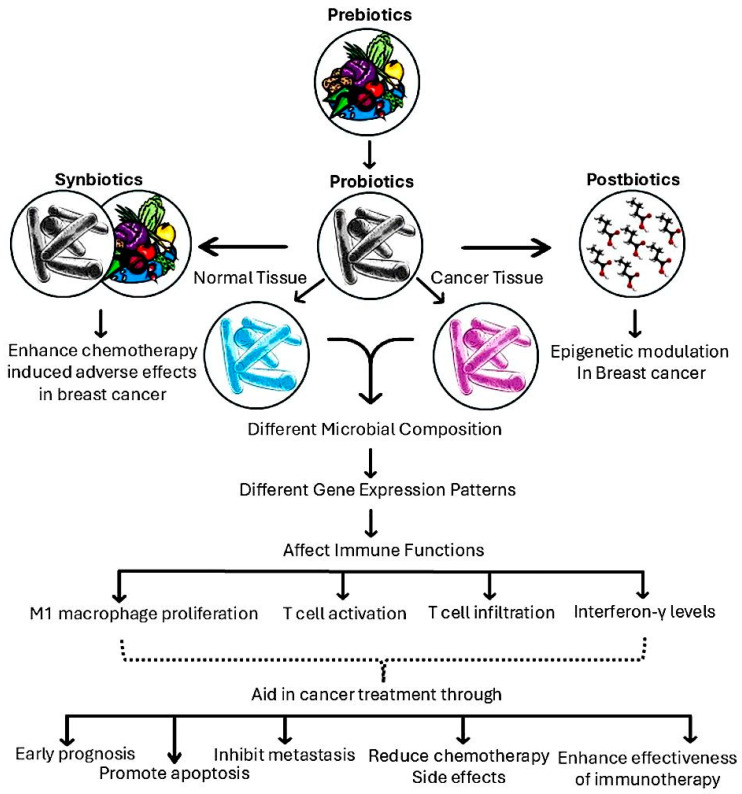
Microbiome modulation in breast cancer treatment. Prebiotics, probiotics, synbiotics, and postbiotics influence breast cancer progression by altering microbial composition, gene expression, and immune functions. Prebiotics support beneficial gut bacteria, while probiotics impact both normal and cancerous tissues. Synbiotics can reduce chemotherapy-induced side effects, whereas postbiotics contribute to epigenetic modulation. These interventions promote macrophage proliferation, T cell activation, and interferon-γ production, aiding in early prognosis, metastasis inhibition, reduced chemotherapy side effects, and improved immunotherapy effectiveness.

In another study, the relationship between intestinal microbiota, chemotherapy side effects, and treatment outcomes was investigated in postmenopausal ER+BC patients undergoing adriamycin, cyclophosphamide, and docetaxel therapy. The study, which included 44 patients who provided 153 stool samples, found a significant decline in microbial richness during treatment, along with notable shifts in bacterial groups such as Proteobacteria, *Lactobacillus*, *Marvinbryantia*, *Christensenellaceae* R7, and *Ruminococcaceae* taxa [141]. Given the essential role of *Christensenellaceae* and *Ruminococcaceae* in short-chain fatty acid production [142], their decrease could disrupt intestinal homeostasis, linking microbiota alterations to chemotherapy-induced side effects. Several mechanisms are proposed (Figure 4).

#### 4.2.3. Intratumoral Microbiota and Metastasis

The significance of intratumoral microbiota in facilitating metastasis has increasingly garnered scholarly interest. In a murine model of breast cancer (MMTV-PyMT), it was observed that bacteria residing within tumors contribute to enhanced metastatic colonization via the reorganization of the actin cytoskeleton in circulating tumor cells, thereby augmenting their resilience against fluid shear stress. The elimination of these bacterial populations resulted in a marked reduction in pulmonary metastasis without influencing the growth of the primary tumor. Moreover, the introduction of specific bacterial strains extracted from tumor-associated microbiota was shown to promote metastasis in various murine cancer models, indicating that targeting tumor-associated microbiota may offer a novel therapeutic strategy [143].

A separate investigation revealed that a high-fat diet (HFD) facilitates breast cancer progression by modifying both gut and mammary microbiota compositions. Fecal transplants from HFD-treated mice to mice on a control diet reproduced these effects, leading to elevated levels of bacterial lipopolysaccharides (LPS), which compromised epithelial permeability in both gut and mammary tissues. The HFD or microbiota derived from HFD-subjected subjects resulted in the downregulation of the genes associated with tight junctions. In contrast, exposure to LPS was correlated with an increased proliferation of breast cancer cells. Furthermore, a clinical trial demonstrated that supplementation with fish oil influenced the microbiota within breast tumors and adjacent normal tissues, underscoring the interrelation between the diet, the microbiota, and breast cancer development [144,145].

#### 4.2.4. Targeting Microbiota to Improve Therapeutic Outcomes

Research has highlighted the potential of microbiota-targeted interventions to enhance breast cancer therapies. In animal models, ampicillin significantly reduced tumor size and inhibited lung metastasis by restructuring the tumor’s immunological environment, promoting M1 macrophage proliferation, T cell infiltration, and reducing microbial recognition pathways. The decline of *Staphylococcus epidermidis* improved the immune response against tumors. Combining ampicillin with paclitaxel further boosted therapeutic efficacy [146].

Another study showed that intratumoral delivery of supernatants from *Bacteroides fragilis* and *Bifidobacterium bifidum* effectively reduced breast tumor development in mice, inducing necrosis and increasing interferon-γ levels while decreasing IL-10. Remarkably, three out of four treated mice became tumor-free within 10–25 days [147].

Additionally, lactic acid bacteria from human breast milk, particularly *P. acidilactici*, decreased MDA-MB-231 breast cancer cell viability in vitro and inhibited cancer cell migration, suggesting its potential as a therapeutic agent [148].

A comprehensive study of 33,780 postmenopausal women over 16.6 years found that high milk consumption increased the risk of estrogen receptor-positive breast cancer. In contrast, a high intake of fermented dairy products was linked to a reduced risk of estrogen receptor-negative breast cancer [149].

Overall, targeting microbiota through therapeutic interventions shows promise as a complementary approach in breast cancer management, emphasizing the need to understand dietary influences on microbiota to tailor effective treatments.

## 5. Translational and Clinical Applications of Prebiotics in BC

### 5.1. The Role of Prebiotics in BC Treatment

Several investigations have explored the potential of prebiotics as adjunctive therapeutic strategies for breast cancer management. Several reports [101,150] have demonstrated that prebiotic supplementation significantly improved microbiota composition and serum biomarkers in breast cancer patients undergoing surgery, chemotherapy, and radiotherapy. These studies have reported enhanced absolute neutrophil count, reduced fasting glucose concentrations, and lower low-density lipoprotein (LDL) cholesterol levels—factors crucial in mitigating breast cancer recurrence.

Microbiome analysis further revealed notable shifts in biological specimens such as feces, serum, and urine, with *Ruminococcus* sp. and *Streptococcus* sp. being the most significantly affected bacterial taxa. Additionally, adverse effects linked to prebiotic consumption were predominantly mild and gastrointestinal, suggesting their safe integration into breast cancer treatment protocols [151].

### 5.2. Synergistic Effects of Prebiotics and Exercise

Recent research has highlighted the potential of combining prebiotic supplementation with exercise to enhance health outcomes in breast cancer patients. While exercise alone induced minimal changes in gut microbiota composition, microbiota transplantation from post-exercise individuals into germ-free mice led to reduced tumor proliferation and improved immune responses, characterized by lower vascular endothelial growth factor (VEGF) levels [152]. The combination of prebiotic supplementation with exercise further enhanced beneficial microbial shifts and strengthened antitumor immune responses, indicating a potential synergistic approach to improving clinical outcomes in breast cancer patients [153].

Physical activity has been shown to influence the gut microbiota composition in breast cancer survivors. In a study where participants engaged in a 12-week exercise program, fecal microbiota transplantation (FMT) from post-exercise individuals into germ-free mice resulted in reduced tumor volume and favorable immune profiles [154]. This suggests that exercise-induced changes in the gut microbiota can have systemic effects, including modulation of tumor progression and immune function.

Prebiotics, such as oligofructose, serve as dietary fibers that selectively stimulate the growth of beneficial gut bacteria. When combined with exercise, prebiotic supplementation has been associated with enhanced antitumor immune responses. A study demonstrated that the combination of prebiotic supplementation with exercise further enhanced beneficial microbial shifts and strengthened antitumor immune responses, indicating a potential synergistic approach to improving clinical outcomes in breast cancer patients [155].

Integrating exercise and prebiotic supplementation into the care regimen of breast cancer patients may offer a non-pharmacological strategy to modulate the gut microbiota and immune system, potentially improving treatment outcomes and the quality of life [156]. However, further clinical trials are necessary to confirm these findings and establish standardized guidelines for implementation.

### 5.3. Prebiotics and Immune Checkpoint Therapy

Fucoidan, a natural dietary compound with prebiotic properties, has been found to potentiate the effects of anti-PD-1 monoclonal antibody immunotherapy by modulating the gut microbiota and the associated metabolites. Fucoidan is a sulfated polysaccharide primarily derived from brown seaweeds such as *Laminaria japonica* and *Undaria pinnatifida*. Recent studies have highlighted its potential as an immunomodulatory agent, particularly when used in combination with immune checkpoint inhibitors, such as anti-PD-1 monoclonal antibodies [157].

The gut microbiota has long been recognized for its significant influence on the immune function, with emerging research indicating its critical role in cancer therapy, particularly in modulating responses to immunotherapy. Fucoidan acts as a prebiotic, influencing the composition and diversity of the gut microbiota. In a study by Li et al. [158], fucoidan supplementation in mice undergoing anti-PD-1 immunotherapy resulted in a significant increase in populations of beneficial gut bacteria, including *Bifidobacterium*, *Faecalibaculum*, and *Lactobacillus*. These bacterial species are known for their immunoregulatory functions, and their enhancement by fucoidan was associated with a notable improvement in immune responses against tumors [158]. This modulation of the gut microbiota represents an exciting avenue for enhancing the effectiveness of immunotherapies.

Fucoidan has a multifaceted role in immune modulation. It has been shown to activate macrophages and dendritic cells, key players in the initiation of immune responses. In animal models, fucoidan has been demonstrated to enhance the proliferation of spleen lymphocytes and peritoneal macrophages, thereby boosting both innate and adaptive immune responses [159]. Additionally, fucoidan supplementation has been associated with increased production of pro-inflammatory cytokines, such as TNF-α and IL-6, which are crucial for sustaining immune responses and improving antitumor activity [160].

The synergy between fucoidan and anti-PD-1 monoclonal antibodies has been investigated in various cancer models. This combination therapy leads to enhanced tumor suppression with a reduction in tumor size and weight, as compared to either treatment alone [161]. The improved efficacy of this combination is thought to arise from the fucoidan-induced modulation of the gut microbiome, which, in turn, optimizes the immune system’s ability to respond to PD-1 blockade. For example, a study by Zhang et al. [162] revealed that fucoidan treatment not only promoted a shift towards a more favorable microbiota, but also increased the population of CD8+ T cells, which are essential for tumor cell destruction in PD-1 inhibition therapies. Furthermore, fucoidan-induced changes in the microbiota led to the increased production of short-chain fatty acids (SCFAs), which are known to activate immune cells and potentiate the effects of immunotherapies [163].

Recent research continues to uncover the complexity of fucoidan’s role in cancer immunotherapy. Yang et al. [164] have demonstrated that fucoidan not only enhances the gut microbiome diversity, but also influences metabolic pathways in tumor-bearing hosts, contributing to a more robust antitumor response when combined with PD-1 inhibitors. The exploration of fucoidan’s impact on the gut microbiome and immune system presents an exciting frontier in cancer therapy, offering a natural adjunctive strategy to optimize immunotherapeutic outcomes.

Overall, the combination of fucoidan with anti-PD-1 monoclonal antibodies represents a promising therapeutic approach, leveraging the gut microbiota’s influence on immune modulation to enhance cancer treatment efficacy. However, further clinical studies and trials are needed to fully understand the mechanisms at play and determine the optimal dosage and treatment regimens for fucoidan in immunotherapy settings.

### 5.4. Gut Microbiota Modulation by Phytochemicals

Sweet potato-derived compounds. A study on three bioactive compounds—daucosterol linolenate (DLA), daucosterol linoleate (DL), and daucosterol palmitate (DP)—from sweet potatoes demonstrated significant tumor suppression in MCF-7 xenograft models. These compounds inhibited tumor growth, reduced serum tumor markers, activated apoptotic pathways, and altered the gut microbiota composition by increasing Bacteroidetes and decreasing Firmicutes, supporting their potential in breast cancer prevention [165].

Fructus Bruceae Oil (BO). Extracted from *Brucea javanica*, BO exhibited breast cancer suppression via gut microbiota modulation and mTOR pathway inhibition. Interestingly, BO lacked efficacy in germ-free conditions, reinforcing the critical role of gut microbiota in its anticancer effects [166].

Ginkgo biloba leaf extract (GLE). GLE was shown to reduce the intestinal breast cancer resistance protein (BCRP) expression, enhancing sulfasalazine bioavailability. Additionally, it altered the gut microbiota composition, suggesting its role in modifying drug transporter activity and herb–drug interactions in breast cancer treatment [167].

Poria cocos ethanol extract. This extract enhanced intestinal barrier integrity and microbiota composition in breast cancer models by upregulating tight junction proteins and promoting beneficial bacterial growth while reducing pathogenic microbes, highlighting the gut microbiota’s role in intestinal and systemic health [168].

*Camellia Sinensis* nanovehicles. Nanovehicles derived from *Camellia sinensis* flowers exhibited anticancer properties by inducing apoptosis and suppressing tumor growth and metastasis, along with modulating gut microbiota composition in preclinical models [169].

### 5.5. The Role of SCFAs in BC Treatment

Short-chain fatty acids (SCFAs), primarily acetate, propionate, and butyrate, are metabolites produced by the gut microbiota through the fermentation of dietary fibers. Beyond their role as energy sources, SCFAs have emerged as significant modulators in cancer therapy, particularly in breast cancer.

SCFAs have demonstrated potential as selective estrogen receptor downregulators (SERDs) in endocrine-resistant breast cancer. Butyrate, propionate, and acetate effectively downregulate both wild-type and mutant estrogen receptor alpha (ERα). Notably, butyrate and propionate also promote histone acetylation, mimicking the effects of histone deacetylase (HDAC) inhibitors, thereby influencing gene expression related to cancer progression [170].

Further research into sodium butyrate (NaB) and sodium propionate (NaP) has demonstrated their ability to inhibit MCF-7 breast cancer cell proliferation in a dose-dependent manner. NaB exhibited greater potency than NaP, inducing cellular differentiation, cell cycle arrest at the G1 phase, and extensive apoptosis at higher concentrations [171].

SCFAs exert their anticancer effects through multiple mechanisms:

Epigenetic modulation. SCFAs influence gene expression by modifying histone acetylation and methylation patterns, leading to the activation of tumor suppressor genes and inhibition of oncogenes [172].

Receptor-mediated signaling. SCFAs interact with free fatty acid receptors (FFARs), such as FFAR2 and FFAR3, on breast cancer cells, modulating pathways that regulate cell proliferation, differentiation, and apoptosis [173].

Immune system modulation. SCFAs influence immune cell function, enhancing the antitumor activity of cytotoxic T lymphocytes and natural killer cells, thereby supporting the body’s immune surveillance against cancer cells [174].

Integrating SCFAs into breast cancer therapy offers a promising strategy to overcome endocrine resistance and support treatment efficacy. Dietary interventions rich in fiber can promote SCFA production, potentially serving as adjunctive treatments alongside conventional therapies. However, further clinical studies are necessary to establish optimal SCFA formulations, dosages, and treatment regimens to maximize therapeutic benefits.

### 5.6. Combination Therapies with SCFAs and Phytochemicals

Recent studies have demonstrated that combining short-chain fatty acids (SCFAs) such as sodium butyrate (NaB) with phytochemicals such as sulforaphane (SFN) and genistein (GE) can significantly enhance therapeutic outcomes in breast cancer. While exercise alone induced minimal changes in the gut microbiota composition, microbiota transplantation from post-exercise individuals into germ-free mice led to reduced tumor proliferation and improved immune responses, characterized by lower vascular endothelial growth factor (VEGF) levels [152]. The combination of prebiotic supplementation with exercise further enhanced beneficial microbial shifts and strengthened antitumor immune responses, indicating a potential synergistic approach to improving clinical outcomes in breast cancer patients [153].

The tri-combination of SFN, NaB, and GE has been shown to exhibit synergistic effects in inhibiting breast cancer cell proliferation. This combination was more effective than individual treatments in reducing cell viability, inducing apoptosis, and arresting the cell cycle at the G2/M phase. Mechanistically, this therapy suppressed the key epigenetic regulators, including DNA methyltransferases (DNMTs), HDACs, and histone methyltransferases, while promoting histone acetylation. These findings highlight the potential of combining dietary components to modulate epigenetic mechanisms for breast cancer prevention and treatment [155].

Sulforaphane (SFN), an isothiocyanate found in cruciferous vegetables, has demonstrated anticancer properties by modulating epigenetic regulators. It inhibits HDACs and enhances histone acetylation, leading to the suppression of tumorigenic genes. SFN also induces apoptosis and cell cycle arrest in breast cancer cells [154]. Sodium butyrate (NaB), a SCFA produced by gut microbiota fermentation of dietary fibers, serves as an HDAC inhibitor. It induces histone acetylation, leading to the activation of tumor suppressor genes and inhibition of cancer cell proliferation [175]. Genistein (GE), an isoflavone abundant in soy products, acts as a DNMT inhibitor. It demethylates promoter regions of tumor suppressor genes, reactivating their expression and inhibiting breast cancer cell growth [176].

Integrating SFN, NaB, and GE into breast cancer therapy offers a promising strategy to modulate epigenetic mechanisms. This combination targets multiple pathways, including DNA methylation, histone modification, and cell cycle regulation, to inhibit tumor progression. Further clinical studies are warranted to validate these findings and establish optimal dosing regimens for effective translation into therapeutic interventions [177].

### 5.7. Synbiotics as Supportive Care in BC Treatment

Synbiotics, which combine probiotics and prebiotics, have been explored as adjunctive therapies to alleviate chemotherapy-related adverse effects.

A clinical trial reported that synbiotic supplementation significantly reduced abnormal defecation and fatigue over an eight-week period. While nausea, vomiting, and anorexia also decreased, these changes were not statistically significant compared to placebo controls [178].

A systematic review of randomized clinical trials found that synbiotics containing *Lactobacillus* and *Bifidobacterium* species, along with prebiotic fructooligosaccharides (FOS), were effective in mitigating obesity and dyslipidemia among breast cancer patients and survivors [179].

A study investigating synbiotic supplementation with caloric restriction in breast cancer-related lymphedema (BCRL) demonstrated that participants in the intervention group showed significant improvements in the quality of life, reductions in edema volume, and lower body mass indices compared to the controls. However, differences between the synbiotic and caloric restriction groups were not statistically significant, suggesting that synbiotics may provide additional, albeit modest, benefits in lymphedema management [180].

Emerging evidence supports the integration of prebiotics, synbiotics, and SCFA-based therapies into breast cancer treatment regimens. Prebiotics not only modulate the gut microbiota, but also influence the immune function, epigenetic regulation, and response to conventional cancer therapies. While clinical studies continue to refine their therapeutic potential, prebiotics and synbiotics hold promise as complementary interventions that may enhance treatment efficacy, reduce side effects, and improve the patients’ quality of life.

## 6. Conclusions

Breast cancer remains a major global health challenge, necessitating the exploration of novel therapeutic strategies to enhance treatment efficacy and improve patient outcomes. Estrogen receptor signaling is a key driver of breast cancer progression, with ERα acting as an oncogene and ERβ exhibiting tumor-suppressive properties. The intricate crosstalk between ER signaling and other molecular pathways, such as PI3K/AKT/mTOR, underscores the need for innovative approaches to target endocrine resistance and tumor progression.

Emerging evidence suggests that prebiotics play a pivotal role in breast cancer prevention and therapy by modulating the gut microbiota composition, influencing systemic estrogen metabolism, and enhancing immune responses. The production of short-chain fatty acids (SCFAs) through prebiotic fermentation has demonstrated significant antitumor effects, including epigenetic regulation, metabolic reprogramming, and suppression of inflammatory pathways. Furthermore, prebiotics and synbiotics have shown promise in alleviating chemotherapy-related side effects and improving the quality of life in breast cancer patients.

Future research should focus on large-scale clinical trials to validate the efficacy of prebiotic interventions in breast cancer management. Personalized approaches that integrate dietary modifications, microbiome profiling, and targeted therapeutics may provide a more comprehensive strategy for improving patient outcomes. The integration of prebiotics into standard cancer care holds great potential for enhancing treatment responses and paving the way for microbiota-based precision medicine in oncology.

## Figures and Tables

**Table 1:** Prebiotic-driven microbiota changes and their immunological impacts in relation to breast cancer (↑ means increase/↓ decrease).

Microbial/Metabolite Change	Immune Effects	Relevance to Breast Cancer	Refs.
↑ ***Bifidobacterium* and *Lactobacillus***	Strengthen the gut barrier, decrease systemic lipopolysaccharides, increase the IL-10 production, and enhance the dendritic cell function.	Reduced chronic inflammation and improved antitumor T cell priming.	[98,99]
↑ **Butyrate-producing bacteria *(Faecalibacterium)***	↑ Butyrate levels: HDAC inhibition in T cells and macrophages; promote Treg differentiation and IL-10; suppress M2 macrophages; activate NK cells.	Anti-inflammatory milieu (less NF-κB activity) potentiates innate tumor killing and could limit metastasis.	[98,99]
↑ **Propionate and acetate**	Via GPR43 on neutrophils and GPR41 on monocytes, modulate chemotaxis and phagocytosis; propionate also inhibits HDAC to reduce Th17 cytokines.	Controlled inflammatory responses (prevent excessive Th17, which can aid tumor angiogenesis); maintain immune surveillance by neutrophils.	[100]
↓ ***Clostridia* and *E. coli* (high GUS producers)**	Lower the microbial antigen load that drives inflammation; possibly reduce TLR activation systemically.	Decreased protumor inflammation (e.g., less IL-6, which can stimulate cancer cell proliferation) may reduce TLR4-mediated tumor growth signals.	[101]
↑ ***Akkermansia muciniphila***	Enhances TLR2 signaling, strengthens the gut barrier, and increases anti-inflammatory cytokines such as IL-10.	Promotes an anti-inflammatory environment and may inhibit tumor progression.	[102]
↑ ***Faecalibacterium prausnitzii***	Produces butyrate, induces Treg differentiation, and suppresses pro-inflammatory cytokines.	Anti-inflammatory milieu; potential reduction in tumor-promoting inflammation.	[103]
↑ ***Bacteroides fragilis***	Polysaccharide A induces IL-10 production and enhances the regulatory T cell function.	Modulates immune response and may reduce inflammation-associated tumorigenesis.	[104]
↑ ***Roseburia* spp.**	Butyrate production enhances mucosal immunity and induces Treg cells.	Supports gut integrity; potential protective role against cancer.	[105]
↑ ***Clostridium butyricum***	Butyrate production promotes Treg cells and inhibits pro-inflammatory cytokines.	Anti-inflammatory effects may suppress tumor growth.	[106]
↑ ***Ruminococcus bromii***	Resistant starch degradation; butyrate production; supports the gut barrier.	Enhances gut health; potential indirect effects on tumor suppression.	[107,108]
↑ ***Eubacterium* sp.**	Produces butyrate and propionate and modulates immune responses.	SCFA-mediated anti-inflammatory effects; possible tumor suppression.	[109,110]
↑ ***Bifidobacterium longum***	Enhances dendritic cell maturation, increases IFN-γ production, and supports NK cell activity.	Boosts antitumor immunity; potential to enhance immunotherapy efficacy.	[111,112]
↑ ***Streptococcus thermophilus***	Produces lactate and modulates macrophage polarization towards the M1 phenotype.	Promotes antitumor immune responses and may inhibit tumor growth.	[113]
↑ ***Veillonella parvula***	Lactate fermentation to propionate modulates T cell responses.	SCFA-mediated immune modulation; potential anticancer effects.	[114]
↑ ***Anaerostipes caccae***	Butyrate production enhances Treg cell differentiation and suppresses inflammation.	Supports an anti-inflammatory environment and may reduce cancer risk.	[115]
↑ ***Coprococcus comes***	Produces butyrate, modulates the gut barrier function, and influences immune homeostasis.	Maintains gut integrity; potential protective role against tumorigenesis.	[116]
↑ ***Desulfovibrio piger***	Produces hydrogen sulfide, modulates inflammatory responses, and impacts the gut barrier.	Complex role: excessive hydrogen sulfide may promote inflammation, while balanced levels could support gut health.	[117]

## Data Availability

All data generated are presented in the current MS.

## Abbreviations

AC: acetate; AD: Alzheimer’s disease; APCs: antigen-presenting cells; ATP: adenosine triphosphate; BBB: blood–brain barrier; BC: breast cancer; BCRP: breast cancer resistance protein; BDNF: brain-derived neurotrophic factor; BMI: body mass index; CDK: cyclin-dependent kinase; CHEK2: checkpoint kinase 2; DNA: deoxyribonucleic acid; DNMTs: DNA methyltransferases; ER: estrogen receptor; ERα: estrogen receptor alpha; ERβ: estrogen receptor beta; ERBB: erythroblastic leukemia viral oncogene homolog; *E. coli*: *Escherichia coli*; EGFR: epidermal growth factor receptor; ESR1: estrogen receptor 1; FAS: fatty acid synthase; FFARs: free fatty acid receptors; FOS: fructooligosaccharides; GE: genistein; GPR: G protein-coupled receptor; GUS: β-glucuronidase; HDAC: histone deacetylase; HER2: human epidermal growth factor receptor 2; HFD: high-fat diet; HR+: hormone receptor-positive; IFN-γ: interferon gamma; IL: interleukin; LPS: lipopolysaccharide; M1: tumoricidal macrophages; M2: tumor-associated macrophages; MAP: mean arterial pressure; MDSCs: myeloid-derived suppressor cells; MMPs: matrix metalloproteinases; mTOR: mammalian target of rapamycin; NaB: sodium butyrate; NaP: sodium propionate; NF-κB: nuclear factor kappa B; NK: natural killer; PD-1: programmed cell death protein 1; PGRMC1: progesterone receptor membrane component 1; PI3K: phosphoinositide 3-kinase; PR: progesterone receptor; PRR14: proline rich 14; PTEN: phosphatase and tensin homolog; RANKL: receptor activator of nuclear factor kappa B ligand; RB1: retinoblastoma 1; R7: *Christensenellaceae* R7; SCFAs: short-chain fatty acids; SERDs: selective estrogen receptor degraders; SIN1: stress-activated map kinase-interacting protein 1; TAMs: tumor-associated macrophages; TGF-β: transforming growth factor beta; Th1: T-helper 1 cells; TNBC: triple-negative breast cancer; TNF-α: tumor necrosis factor alpha; Treg: regulatory T cell; VEGF: vascular endothelial growth factor; Wnt: wingless/integrated.
